# Risk and protection factors of mental stress among medical staff in the third year of the COVID-19 pandemic

**DOI:** 10.3389/fpsyt.2024.1334552

**Published:** 2024-03-22

**Authors:** Christiane Eichenberg, Raphaela Schneider, Phillip Auvera, Gabor Aranyi, Kurt Huber

**Affiliations:** ^1^ Faculty of Medicine, Institute of Psychosomatics, Sigmund Freud Private University, Vienna, Austria; ^2^ Sigmund Freud Private University, Medical Faculty, Vienna, Austria; ^3^ Faculty of Psychotherapy Science, Sigmund Freud Private University, Vienna, Austria; ^4^ Institute of Education and Psychology at Szombathely, ELTE Eötvös Loránd University, Budapest, Hungary; ^5^ 3rd Dept. of Medicine, Cardiology and Internal Intensive Care Medicine, Clinic Ottakring (former Wilhelminenhospital), Vienna, Austria

**Keywords:** COVID-19 pandemic, risk factor, mental stress, medical staff, anxiety, PTSD, posttraumatic stress symptoms

## Abstract

**Background:**

The COVID-19 pandemic placed an extraordinary burden on health care workers (HCW), who are reported to suffer from great mental stress. The current study investigates the mental health of HCW in the later phases of the pandemic.

**Methods:**

HCW completed the following questionnaires online (06/2021-02/2022, *N*=159): demographics (age, gender, profession, ward), Impact of Event Scale (IES-R, posttraumatic stress), State Trait Anxiety Inventory (STAI-S, state anxiety), stress-coping questionnaire (SVF-78), and bespoke corona-specific stress and protective-factor questions (5 items each). We used factor analysis to test scale properties and regression-type methods (t-tests, ANOVA, multiple regression) for hypothesis tests and effect-size estimation.

**Results/discussion:**

Mental stress in HCW is influenced by similar factors as described for earlier phases. However, differences to earlier phases were found in ward affiliation which is no longer a variable of concern for explaining differences in mental health of HCW. Further, even if nurses are the occupational group with the highest mental stress as in prior research, detailed analysis shows that medical specialists with close proximity to patients with a high-level of responsibility are the most burdened sub-group. Unlike nurses, they suffer from high levels of anxiety in addition to high levels of post-traumatic and COVID-specific stress. Analyses showed further that COVID-specific stress is the strongest predictor of mental stress, wherein COVID-specific stress factors remain the same as reported in literature on the early pandemic phases. HCW showed to use still more positive than negative coping strategies. Negative strategies increased as expected mental stress, whereas positive strategies alleviated only anxiety. Additionally, we found that doctors benefited from many protective factors while nurses had access to fewer protective factors like earlier waves.

**Conclusion:**

Data show that HCW still suffer from mental stress in the third year of the pandemic. HCW of all hospital wards may be affected by mental stress and need attention and protective measures. Medical specialists are the most burdened subgroup. Detailed analyses show that properties other than occupation, gender, or ward affiliation are more appropriate to evaluate mental stress of HCW. The findings have implications for developing specialized protection strategies for the post-pandemic phase and future pandemics.

## 1 Introduction

The COVID-19 pandemic has imposed an extraordinary burden on healthcare workers (HCW), pushing them to their professional and personal limits. Consequently, many have either expressed the desire to resign or have already done so (1). In a literature review conducted by De Pablo et al. (2), it was demonstrated that HCW exposed to COVID-19 during the early stages of the pandemic experienced various mental health issues, including anxiety, post-traumatic stress, and psychological distress. Italian HCW reported significant concerns regarding the potential transmission of the virus to themselves and their family members during the initial wave of the pandemic (3). A qualitative interview study conducted in the second year of the pandemic revealed that HCW continued to grapple with fears of infection, mental distress, moral dilemmas, and interpersonal conflicts among colleagues (4). Evidence suggests that even in later stages of the pandemic, the mental health of HCW remains as challenging as observed in the preceding waves. For instance, Arab HCW reported a lower quality of life in the third year of the pandemic (5). Pei et al. (6) observed substantial mental health issues among Chinese HCW working in the isolated environment of square cabin hospitals during the same period. Th’ng et al. (7) reported consistently poor mental health among HCW across all three years of a longitudinal study.

Not all HCW react to pandemic-related stress in the same way, as responses to disasters can vary significantly between individuals (8). Eichenberg et al. (9) demonstrated based on the health belief model during the early stages of the COVID-19 pandemic that various socio-demographic and personality-specific factors influence adherence to COVID-related preventive measures and subsequent pandemic-specific health behaviors. Previous pandemics have identified factors associated with more severe mental health issues, including female gender, younger age, less job seniority, frontline work, and the nursing profession (10). During the COVID-19 pandemic, a multitude of factors have been identified as stressors (1). For example, nursing staff and female HCW (3, 11–14) as well as frontline healthcare workers (3, 12), face an elevated risk of developing mental stress compared to other healthcare workers. Additionally, the fear of infection increases the susceptibility to mental health issues (3, 15). In contrast to these findings, Pei et al. (6) observed no statistically significant differences in mental health problems during the third year of the pandemic concerning gender, job status, age, or job position among HCW.

Kramer et al. (1) inferred from the literature that in the majority of the studies, more than 50% of HCW experienced clinically significant anxiety levels. Among these, the groups most affected were nurses and women (1). An umbrella review conducted by Dragioti et al. (16), which encompassed 44 Meta-analyses between 2020 and 2021, concluded that 29.9% of HCW suffered from anxiety. Research findings regarding the magnitude of anxiety during the pandemic present contradictions. Van Steenkiste et al. (17) found that anxiety levels decreased over time between April 2020 and June 2020 among ICU HCW. In contrast, a larger longitudinal study spanning 4 to 5 months until September 2020 found that mental health issues, including anxiety, worsened with time (18). This deterioration was primarily linked to concerns about infection.

Van Steenkiste et al. (17) established a strong connection between distress in the first year of the pandemic and PTSD. In their umbrella review, Dragioti et al. (16) summarized that 18.75% of HCW experienced PTSD. Literature from the initial COVID-19 wave demonstrated a high prevalence of PTSD symptoms, particularly among older HCW (19), women, nurses, and frontline workers (10, 19, 20). For the early phase of the pandemic, the fear of contracting COVID-19 emerged as the best predictor for anxiety and post-traumatic stress (3). Canal-Rivero et al. (21) found that over six months in 2020, post-traumatic stress improved in female HCW but worsened in males.

A meta-synthesis of studies on resilience factors of HCW in a pandemic synthesized studies between 2002 and 2022, including COVID-19, H1N1, MERS, EBOLA, and SARS (22)(Curtin et al., 2022). Overall factors for HCW resilience were professional identity, collegial support, effective communication from supportive leaders, and the possibility to engage in self-care and experiences of growth (22). For COVID-19, a literature review concluded that HCW showed high to moderate levels of mental resilience as a protective factor for HCW in the first year of the pandemic (23). Lin et al. (24) reported lower levels for nurses compared to doctors. Labrague (23) also investigated coping strategies in HCW during the early phase of the COVID-19 pandemic and found that HCW used more positive than negative strategies. Thus, HCW have access to their resource of positive coping when confronted with the stress of a pandemic. Support from and communication with family, friends, and colleagues, religious behavior, distraction activities, and understanding COVID-19 medically connected with adhering to safeguard measures are the most reported coping mechanisms found in the literature (23). In their literature review, Labrague et al. (23) summarized that positive coping strategies decrease, whereas negative coping strategies increase levels of mental health problems. Additionally, adaptive coping mechanisms were found to influence the well-being of Scottish health care workers also during the further course of the pandemic (25).

The COVID-19 pandemic remains as an ongoing and exceptional stress event that has deeply impacted individuals on a global scale. Based on the stress-coping model (26), individuals face stressors and use coping mechanisms throughout life. Mental well-being depends on balancing the demands of life events and the availability of coping strategies to manage them (26). In light of this, our study delves into both the stressors faced by HCW during the pandemic and the diverse coping mechanisms they employ. Since HCW bear a significant mental burden for society during a pandemic, and considering the existing protective measures, it becomes imperative to comprehend the intricate interplay of factors influencing their mental health throughout the COVID-19 pandemic at different points in time. This understanding can help describe changes and infer tailored protective measures for specific groups of HCW. Such insights are not only crucial for preparing for future pandemics but also for providing differentiated support during the post-pandemic phase. Furthermore, considering altered external conditions in the third year, such as the presence and widespread distribution of vaccinations, the question arises whether COVID-specific stress continues to exert the same level of impact on mental health of HCW.

Considering the aforementioned findings, we posit the following hypotheses:

1. The mental stress experienced by HCW during the later stages of the COVID-19 pandemic varies based on the type of patient care required and proximity to COVID-19, operationalized by ward affiliation. Stress levels are expected to be higher in cases with more challenging patients (Hypothesis 1).
2. The level of mental stress differs also depending on the occupation group (Hypothesis 2) and gender (Hypothesis 3). Stress is anticipated to be higher among nurses and women.
3. We further hypothesize that anxiety and post-traumatic stress correlates positively with the level of COVID-specific stress (Hypothesis 4). Higher levels of COVID-specific stress are expected to correspond to elevated mental stress.
4. This study aims to test the Hypothesis that coping strategies employed by HCW impact their mental health. Positive strategies are predicted to alleviate mental stress, while negative strategies are predicted to exacerbate it (Hypothesis 5).

Additionally, as part of this study, the potential influence of protective factors related to COVID-specific stress on coping with anxiety and post-traumatic stress will be investigated in an explorative way.

## 2 Materials and methods

### 2.1 Data collection

Data were collected using an online survey of medical staff of the Clinic Ottakring, Vienna, Austria (collection period: 06/2021-02/2022). All data was collected in German language. The questionnaire was administered directly or electronically for the medical staff. After providing informed consent and completing the survey, participants were directed to a debriefing page that fully described the study goals and provided the researchers’ contact details if they wanted support for the topics covered or had further questions. The survey was conducted anonymously and in compliance with the data protection guidelines. Participation was not part of the clinic work schedule of the participants but was done on a voluntary basis. Respondents received no reimbursement for participation.

The survey was reviewed and approved by the Ethics Committee of the Faculty of Psychotherapy Science and the Faculty of Psychology of the Sigmund Freud Private University (Reference: UBRPHK9TAQOGJ888066). Given the sensitive nature of this topic, maintaining the privacy of responses was a key part of the engagement strategy. Therefore, measures were employed to ensure anonymity, namely: name and email addresses being linked only to an anonymized personalized ID, IP addresses were not saved, and cookies were not set. Participants could withdraw from the research at any time and have their data deleted at their request, by emailing a researcher and citing their code, allowing for the identification of their anonymized data.

### 2.2 Inclusion criteria

Medical staff of one of the three corona specific wards (normal ward, AIRVO: non-invasive ventilation ward, and ICU: intensive care unit) were used for participation. All adult subjects with a minimum age of 18 years who were assigned to one of the three wards during the survey period were included. There were no specifications regarding a maximum age. In addition to women and men, those who classified themselves as none of the two were also admitted. Medical staff are defined as doctors and nurses who are chief doctors, senior doctors, specialists, ward doctors, interns, assistant doctors, nurses, or other medical staff. Only those individuals were included in the data processing who answered the questionnaire comprehensively.

### 2.3 Survey structure

The survey was created with the SoSci Survey online survey tool (https://www.soscisurvey.de). It began with a brief, which stated the reason for the research and the aim of the survey. Participants consented to the privacy policy of the study via a checkbox. Section 1 asked about socio-demographic factors and study specific factors including: age, gender, profession, ward affiliation, etc. In Section 2, standardized questionnaires were used to examine anxiety, post-traumatic stress, and stress coping strategies. For COVID-specific distress and protective factors, we deduced items from the literature.

### 2.4 Impact of event scale - revised

The German version of the original IES-R (27) measures typical reactions on extremely stressful events asking with 22 items the subjective response to a specific traumatic event on three subscales: avoidance, intrusion, and hypervigilance. Participants are asked to remember the most stressful event they had suffered and rate how often they have experienced each symptom in the past seven days with a 4-point scale with anchor points (in the German version ranging from 0 to 5: 0 = not at all, 1 = rarely, 3 = sometimes, 5 = extremely). Thus, the total IES-R score ranges between 0 - 110. Maercker and Schützwohl (28) report for the German version good internal consistency (Cronbach’s α) for the scale intrusion: α = .90, the scale avoidance: α = .79, and the scale hypervigilance: α = .90 and moderate to good test–retest reliability after three months (intrusion: *r*tt = .80, avoidance: *r*tt = .66, hypervigilance: *r*tt =.79). They report also sufficient construct validity correlating the IES-R scales with the according symptom sum scores of the ‘Diagnostisches Interview bei Psychischen Störungen’ (DIPS, structured diagnostic interview to assess the most relevant mental disorders according to DSM-IV) showing the following intercorrelations: *r* = .53 (avoidance), *r* = .59 (intrusion), and *r* = .72 (hypervigilance).

### 2.5 State trait anxiety inventory

Originally based on the State-Trait Anxiety Inventory (STAI), which was developed by Charles Spielberger et al. (30), Laux et al. (29) presented a widely used German translation of it. Anxiety is viewed as both a trait as well as a state. Here, considering the focus on the actual pandemic situation participants filled out only the state inventory. These 20 items are formulated as statements on current anxiety with 10 positively and 10 negatively worded items (introduction question for state anxiety: “How do you feel now…”; rated on a 4-point Likert scale ranging from 1 = not at all, 2 = a little, 3 = quite, 4 = very much so) resulting in a sum score between 20 and 80. Laux furthermore tried to consider a balance between emotional and cognitive anxiety variables (29). STAI-S has a good reliability (*r*tt = .90 (31);. Retest reliability for state anxiety is, as expected, lower but remains satisfactory (*r*tt = .43 (31);. The state inventory shows also good validity (specificity and consistence coefficients; 33).

### 2.6 Stress-coping questionnaire SVF-78

The SVF-78 – a short version of the German coping questionnaire SVF-120 - is an established and widely used questionnaire in German speaking countries (32). It measures general coping strategies as general trait (33, 34) consisting of 78 items rated on a 5-point Likert scale (0=not at all, 1=barely, 2=possibly 3=likely and 4=very likely). It is separated in the following 13 subscales: 1. self-aggrandizement by comparison with others, 2. denial of guilt, 3. distraction, 4. substitute gratification, 5. situation control, 6. reaction control, 7. positive self-instructions, 8. need for social support, 9. avoidance, 10. escape, 11. rumination, 12. resignation, and 13. self-blame. These subscales are divided in three main strategies, namely positive (subscales 1-7) negative (10–13), and neutral coping strategies (8, 9) by adding scores of the relevant subscales. High scores for positive coping strategies indicate high levels of strategies that are likely to reduce stress, whereas high scores for negative coping strategies indicate high levels of strategies that are more likely to increase stress. Neutral strategies can entail positive as well as negative consequences. The SVF-78 is well evaluated (34), wherein its validity has been proven by factorial analyses and correlations with divergent and convergent factors (34).

### 2.7 COVID-specific mental stress and protective items

As no standardized test procedure was available at the time of the survey, individual aspects of COVID-related stress and protective factors that could help them cope with that stress were deduced from literature on mental stress in the early stages of the pandemic or from general psychological literature. The items and their answer format are listed in **Table 1**.

**Table 1:** Stress items and protective factors.

Item	Perceived psychological distress (Stress 5)
Stress 1	I’m worried about my health (1, 3).
Stress 2	I fear that I could endanger my immediate environment through my increased risk of infection (3, 8).
Stress 3	With the COVID-19 pandemic, I am more concerned about my patients than usual (e.g., due to difficult case planning (35),; due to high sickness rate amongst HCW or a lack of ventilators (36).
Stress 4	I feel overwhelmed with my current workload (1).
Stress 5	I fear that the COVID-19 pandemic and the stresses associated with it will continue for a longer time (this factor was defined as anticipated load expectation).
	Protective factors (Protect 5)
Protect 1	I feel supported by my employer (22).
Protect 2	My immediate environment (partner, relatives, friends) gives me support for the exercise of my profession (23).
Protect 3	I like to do my job (22).
Protect 4	I am convinced that I am doing a good job (22, 37, 38).
Protect 5	I am happy with my work-life balance.

Each statement was evaluated using a 5-point Likert-scale with the following verbal anchors: 1 = not true at all, 2 = rather not true, 3 = even, 4 = rather true, 5 = is fully true.

### 2.8 Participants

A full survey of HCW of the Clinic Ottakring, Vienna (*N* = 170, response rate = 93.53 %) resulted in 159 participants (103 women [65%], 53 men [33%], 3 other [2%]; mean age = 41.18 years, *SD* = 11.66, range = [22; 65]); 85 worked at the normal ward, 52 worked at the intensive care unit (ICU), and 22 worked at the non-invasive ventilation (AIRVO) station. The occupational distribution of respondents is presented in **Table 2**. We collapsed job categories to doctor/nurse for comparing these two occupational groups. The gender distribution within the collapsed job categories is presented in **Table 3**.

**Table 2:** Number of cases in each job category.

Job	*n*	(%)	Job collapsed	*n*	(%)
Nurse	87	(54.7)	Nurse	87	(59.2)
Other medical	10	(6.3)	Doctor	60	(40.8)
Senior doctor	19	(11.9)			
Specialist doctor	18	(11.3)			
Chief doctor	3	(1.9)			
Resident doctor	20	(12.6)			
Medical student	2	(1.3)			
TOTAL	159		TOTAL	147	

All doctors were collapsed into one category; Medical students and Other medical were excluded from the collapsed categories.

**Table 3:** Gender distribution of job categories.

Job	Women	Men	Other (gender)
Nurse	61	25	1
Doctor	32	27	1
Other (job)	10	1	1
TOTAL	103	53	3

### 2.9 Data analysis

Statistical analyses were conducted in R (version 4.3.0) (39), with the packages ‘lavaan’ (version 0.6-15) (40) for confirmatory factor analysis and ‘psych’ (version 2.3.3) (41) for principal component analysis. OLS regressions and pairwise comparisons were conducted using the base R ‘stats’ package. We used an alpha level of 0.05 for each statistical test (exact *p* values are reported) with Bonferroni-corrected *p* thresholds in multiple comparisons. For pairwise comparisons, we used Levene’s test to check the assumption of equality of variances; however, we report Welch’s tests with degrees of freedom adjusted for unequal variances (42). Shapiro-Wilk tests were used for checking normality with a *p* <.05 criterion for assumption violation. We also report Wilcoxon signed-rank test results where the normality assumption was violated. For interpreting effect sizes, we follow Cohen’s (43) rules of thumb for the following measures: correlation coefficient (*r*) and Cramer’s *V*: 0.1 – small, 0.3 – medium, 0.5 – large; Cohen’s *d*: 0.2 – small, 0.5 – medium, 0.8 – large.

## 3 Results

### 3.1 Scale properties

*STAI-S.* We tested the unidimensionality of STAI-S by conducting confirmatory factor analysis with a robust weighted least squares (WLSMV) to suit the categorical and non-normally distributed nature of the STAI-S responses (44). The unidimensional model was an acceptable fit: χ^2^ (117) = 365.500 (scaled), *p* <.001, χ^2^/df = 2.15 [<.3 – good; see (45)], robust CFI = 0.969 (> 0.95), robust TLI = 0.965 (> 0.95), robust RMSE = 0.066 (slightly above 0.06), SRMR = 0.084 (slightly above 0.08) (see (46), for the interpretation of measures of fit). Internal consistency was high: Cronbach’s alpha = .93. Based on these, we used the sum of item scores to derive the STAI-S metric.

*IES-R*. The unidimensionality of IES-R was tested the same way as that of STAI-S. The one-dimensional model was an acceptable fit: χ^2^ (209) = 368.761 (scaled), *p* <.001, χ^2^/df = 1.76 (<.2 – excellent), robust CFI = 0.965 (> 0.95), robust TLI = 0.961 (> 0.95), robust RMSE = 0.073 (slightly above 0.06), SRMR = 0.087 (slightly above 0.08). Internal consistency was high: Cronbach’s alpha = .93. A three-factor model with the sub-scales Intrusion, Avoidance, and Hyperarousal resulted in a statistically significantly better fit than the unidimensional model, χ^2^ (3) = 29.42, *p* <.001. Model fit: χ^2^(206) = 308.820 (scaled), *p* <.001, χ^2^/df = 1.50 (<.2 – excellent), robust CFI = 0.979 (> 0.95), robust TLI = 0.977 (> 0.95), robust RMSE = 0.057 (< 0.06), SRMR = 0.073 (< 0.08). The internal consistency of each sub-scale was high (alphas = .88,.83, and.84, respectively). We calculated sub-scales by summing each corresponding item and derived an overall IES measure according to Maercker and Schützwohl (27), with cases reaching or exceeding the IES value 0 flagged as risk high risk (henceforth referred to as PTS)^1^.

*Stress 5*. The 5 bespoke stress items (**Table 1**) were treated as reflective indicators (see (46)) of psychological distress among health-care workers specific to the context of the study. To test the unidimensionality of these items, we conducted an exploratory factor analysis with principal component extraction. Despite the low sample size, factorability measures (42) were adequate: Bartlett test: χ^2^(10) = 160.318, *p* <.001; determinant of the r matrix (multicollinearity) = 0.3567 (>.00001); KMO (sampling adequacy): mediocre degree of common variance (overall = 0.725; individual = [0.690; 0.805]). Both Kaiser’s criterion and the visual inspection of the scree plot indicated the extraction of a single factor which accounted for 48% of variance in the indicators, with loadings ranging from.57 to.80. The internal consistency of the scale was adequate: Cronbach’s alpha = .73, 95% CI = [.65;.79]. Based on these results, we averaged the five indicators to derive the Stress 5 metric.

### 3.2 Hypothesis 1: differences in mental stress between wards

The descriptive statistics of mental distress metrics across each ward is presented in **Table 4**. We found no statistically significant difference between the stations (AIRVO/ICU/Normal) in average STAI-S scores (*F*(2,156) = 0.355, *p* = .702, *ns*), mean IES-R score (*F*(2,156) = 0.441, *p* = .644, *ns*) and COVID-specific stress (Stress 5, *F*(2,156) = 0.739, *p* = .479, *ns*). Additionally, ward was not statistically significantly associated with the proportion of people with PTS (IES > 0), χ^2^(2) = 0.158, *p* = .924.

**Table 4:** Descriptive table of stress levels across stations.

Station	*n*	PTSD	PTS prop.	IES mean	*IES* SD	STAI mean	*STAI* SD	Stress 5 mean	*Stress 5* SD
AIRVO	22	4	18.18	-1.65	1.60	40.95	10.67	18.68	3.63
ICU	52	9	17.31	-1.81	1.74	39.27	10.91	18.35	3.65
Normal	85	13	15.29	-1.99	1.67	40.73	10.45	17.71	4.24

PTS, number of people with IES > 0 (PTSD criterion, indicating post-traumatic stress); PTS prop., proportion of people within each station with PTS > 0.

### 3.3 Hypothesis 2: differences in mental stress between occupational groups

Descriptive statistics of psychological distress across categories of occupation and ward are presented in **Table 5**.

**Table 5:** Descriptive table of psychological distress across occupation categories (Nurse/Doctor), across all wards and separately by each station (AIRVO/ICU/Normal).

Station	Job	*n*	PTS	IES mean	*IES* SD	STAI mean	*STAI* SD	Stress 5 mean	*Stress 5* SD
Overall	Nurse	87	20	22.99	-1.51	1.65	38.46	9.73	19.07
	Doctor	60	5	8.33	-2.36	1.59	43.32	11.77	16.53
AIRVO	Nurse	16	4	-1.18	1.41	40.44	10.05	19.19	3.58
	Doctor	6	0	-2.89	1.52	42.33	13.11	17.33	3.72
ICU	Nurse	36	8	-1.49	1.76	38.36	10.70	19.08	3.47
	Doctor	13	1	-2.58	1.57	42.77	11.93	15.85	3.08
Normal	Nurse	35	8	-1.67	1.66	37.66	8.64	19.00	3.73
	Doctor	41	4	-2.21	1.62	43.63	11.82	16.63	4.54

PTS represents the number of participants with IES score > 0 (PTSD risk).

*STAI-S*. Doctors had a statistically significant higher average STAI-S score (*M* =43.32, *SD* = 11.77) than nurses (*M* = 38.46, *SD* = 9.73), *t*(110.85) = -2.635, *p* <.01, *d* = 0.46, 95%CI = [0.13; 0.79] (small). (Normality assumption violated for both nurses and doctors; Wilcoxon test result for the above: *W* = 1934.5, *p* <.01.)

*IES-R*. Nurses had a statistically significant higher average IES-R score (*M* = -1.51, *SD* = 1.65) than doctors (*M* = -2.36, *SD* = 1.59) (note: higher IES means more risk of PTS), *t*(129.89) = 3.137, *p* <.01, *d* = -0.53, 95%CI = [-0.86; -0.19] (medium). (Normality assumption violated for both nurses and doctors; Wilcoxon test result for the above: *W* = 3386, *p* <.01.)

*Overall stress (Stress 5)*. Nurses reported statistically significant higher overall stress level (*M* = 19.07, *SD* = 3.55) than doctors (*M* = 16.53, *SD* = 4.16), *t*(113.66) = 3.854, *p* <.001, *d* = 0.666, 95%CI = [0.325; 1.006] (medium). (Normality assumption violated for nurses; Wilcoxon test result for the above: *W* = 3493.5, *p* <.001.).

*PTS*. There was a statistically significant association between Job (Nurse/Doctor) and PTS (IES > 0; Yes/No), χ^2^(1) = 5.403, *p* = .020, *V* = 0.192 (small). The odds of PTS (IES score > 0) for nurses (0.3) were 3.28 times higher than those for doctors (0.09).

Given the substantial variability observed in mental stress variables across different doctor categories, a decision was made to dissect the job category “doctor” into distinct subcategories, namely senior doctors, residents, and specialists. There was a statistically significant effect of job categories collapsed in terms of seniority on COVID-specific stress, *F*(3, 143) = 10.113, *p* <.001, η^2^ = .175. *Post-hoc* tests with Tukey correction showed nurses (*M* = 19.069, *SD* = 3.553) had a statistically significant higher average level of COVID-specific stress than senior doctors (*M* = 14.273, *SD* = 4.421), *t*(143) = 5.479, *p* <.001, *d* = 1.307 (large). Furthermore, resident doctors (*M* = 17.600, *SD* = 3.102) also had higher COVID-specific stress than senior doctors, *t*(143) = 2.936, *p* = .020, *d* = 0.907 (large). Finally, specialist doctors (*M* = 18.111, *SD* = 3.802) had higher COVID-specific stress than senior doctors, *t*(143) = 3.292, *p* = .007, *d* = 1.046 (large). We conclude that senior doctors had lower COVID-specific stress than any other job category with large effect size, while specialist doctors, residents, and nurses did not differ from one another in terms of COVID-specific stress (all other contrasts had small effect-size and were not stat. sig.).

For anxiety, there was a statistically significant effect of job categories collapsed in terms of seniority, *F*(3, 143) = 5.885, *p* <.001, η^2^ = .110. *Post-hoc* tests with Tukey correction showed nurses (*M* = 38.46, *SD* = 9.73) had a statistically significantly lower average level of anxiety than residents (*M* = 45.35, *SD* = 10.99), *t*(143) = 2.689, *p* = .040, *d* = 0.667 (medium). Furthermore, nurses had a statistically significantly lower average level of anxiety than specialists (*M* = 47.61, *SD* = 13.76), *t*(143) = 3.420, *p* = .004, *d* = 0.886 (large). Finally, nurses did not differ in anxiety from senior doctors (*M* = 37.96, *SD* = 8.76), *t*(143) = 0.205, *p* = .997, *d* = 0.049. Further, specialists were stat. sig. more anxious than seniors, *t*(143) = 2.941, *p* = .020, *d* = 0.935 (large), but they did not differ from residents *t*(143) = 0.674, *p* = .907, *d* = 0.219. We conclude that specialists had higher state anxiety than any other job category with large effect size, closely followed by residents with high scores of anxiety, building with specialists a group of high anxious HCW. Whereas nurses and seniors build a group of low anxious HCW during the pandemic, with no stat. sig. difference within the groups. Residents did not differ stat. sig. from seniors, but there was a statistical tendency in that direction (*t*(143) = 2.317, *p* = .099, *d* = 0.716).

For post-traumatic stress, there was a statistically significant effect of job categories collapsed in terms of seniority, *F*(3, 143) = 5.476, *p* = .001, η^2^ = .103. *Post-hoc* tests with Tukey correction showed nurses (*M* = - 1.51, *SD* = 1.65) had a statistically significant higher average level of post-traumatic stress than residents (*M* = - 2.75, *SD* = 1.29), *t*(143) = 3.117, *p* = .012, *d* = 0.773 (medium) and senior doctors (*M* = - 2.66, *SD* = 1.54), *t*(143) = 3.012, *p* = .016, *d* = 0.719 (medium), whereas nurses do not differ stat. sign. from specialists (*M* = - 1.56, *SD* = 1.74), *t*(143) = 0.126, *p* = .999, *d* = 0.033. Here regarding post-traumatic stress, residents and seniors build a group of HCW with low level of post-traumatic stress during the pandemic, with no stat. sign. difference within the groups, and nurses and specialists build a group of HCW with high level of post-traumatic stress during the pandemic, with no stat. sig. difference within the groups. Specialists do not differ stat. sig. from seniors and residents, but tend in that direction (seniors: *t*(143) = 2.159, *p* = .140, *d* = 0.686, residents: *t*(143) = 2.279, *p* = .108, *d* = 0.740).

### 3.4 Hypothesis 3: gender differences in mental stress

Descriptive statistics of mental distress across gender are presented in **Table 6**. We found no association between gender (women/men) and proportion of people with PTS, χ^2^(1) = 0.052, *p* = .820.

**Table 6:** Descriptive table of psychological distress across gender.

Gender	*n*	PTSD	PTS prop.	IES-R mean	IES-R *SD*	STAI-S mean	STAI-S *SD*	Stress 5 mean	Stress 5 *SD*
Women	103	17	16.50	-1.62	1.56	40.03	10.34	18.58	3.59
Men	53	8	15.09	-2.36	1.79	40.94	11.37	16.96	4.49

3 cases with unspecified gender removed. PTS, number of people with IES > 0 (PTSD criterion, indicating post-traumatic stress); PTS prop., proportion of people within each station with PTS > 0.

We tested the effect of gender (women/men) and occupation type (nurse/doctor) on STAI-S, IES-R, and Stress 5 together. Both predictors were coded as binary antecedents with nurse and female as base categories (nurse = 0, doctor = 1; women = 0, men = 1). As the interaction effect of the antecedents was not statistically significant for any of the criterion variables, we report only simple effects.^2^

**Figure 1:**
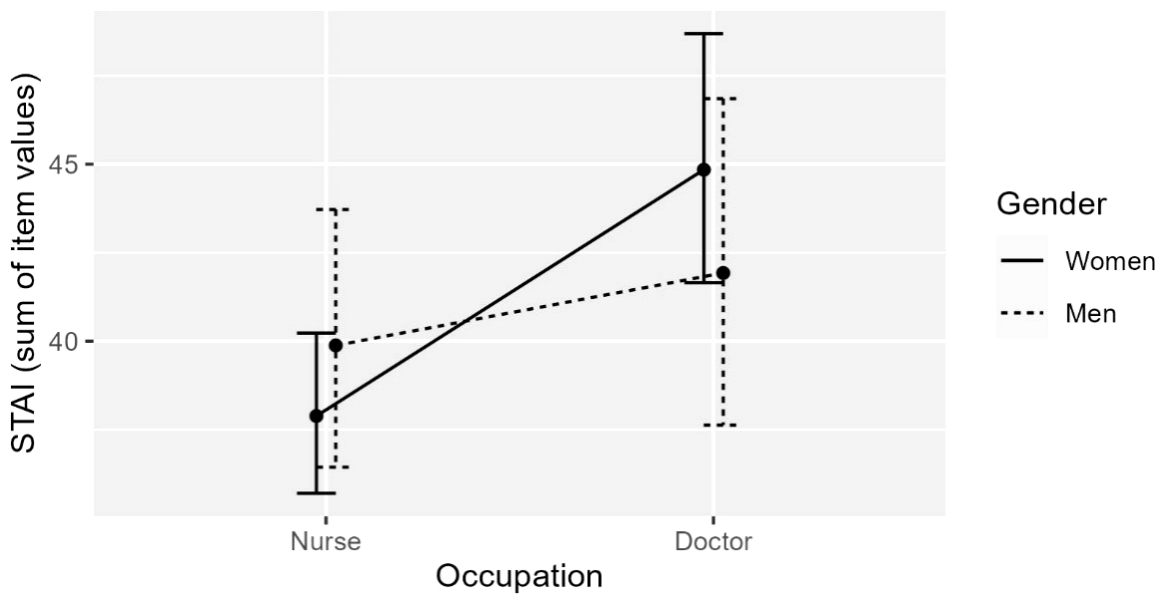
The effect of Gender (women/men) and Occupation (doctor/nurse) on STAI-S score. Only the effect of Occupation stat. sig.; no statistically significant interaction.

*STAI-S*. Only the effect of occupation was a statistically significant predictor of STAI-S (**Figure 1**, see **Table 7**, Panel 1, Model 1), *F*(2,142) = 3.912, *p* <.05, adj. *R* ^2^ = .039: doctors had statistically significant higher mean STAI-S score than nurses, *b* = 5.081, *t*(142) = 2.774, *p* <.01, while the effect of gender was not stat. sig., *b* = -0.227, *t*(142) = -0.121, *p* = .904, *ns*.

**Table 7:** Hierarchical regression analyses predicting anxiety and PTS from COVID-specific stress, controlling for gender and occupation.

*Panel 1*. Outcome variable: anxiety (STAI-S)				
	Model 1		Model 2	
	*b*	β	*b*	β
Intercept	38.531***		21.206***	
Gender	-0.227	-0.021	0.945	0.087
Occupation	5.081**	0.467	7.107***	0.653
Stress 5			0.893***	0.328
*R* ^2^	0.052		0.147	
Adjusted *R* ^2^	0.039		0.129	
AIC	1103.160		1089.912	
*F* statistic	*F*(2,142) = 3.912*		*F*(3,141) = 8.089***	
*F*(Model1, Model2)			*F*(1,141) = 15.635***	
*Panel 2.* Outcome variable: PTS (IES-R)				
	Model 1		Model 2	
	*b*	β	*b*	β
Intercept	-1.338***		-3.541***	
Gender	-0.647*	-0.389	-0.498	-0.299
Occupation	-0.689**	-0.414	-0.431	-0.259
Stress 5			0.114***	0.272
*R* ^2^	0.090		0.155	
Adjusted *R* ^2^	0.077		0.137	
AIC	552.840		544.037	
*F* statistic	*F*(2,142) = 6.986***		*F*(3,141) = 8.617***	
*F*(Model1, Model2)			*F*(1,141) = 10.906**	

*: *p* <.05; **: *p* <.01; ***p <.001. *N* = 145 for each model. Betas for binary predictors (gender and occupation) are partially standardized regression coefficients, and fully standardized coefficients for Stress 5.

**Figure 2:**
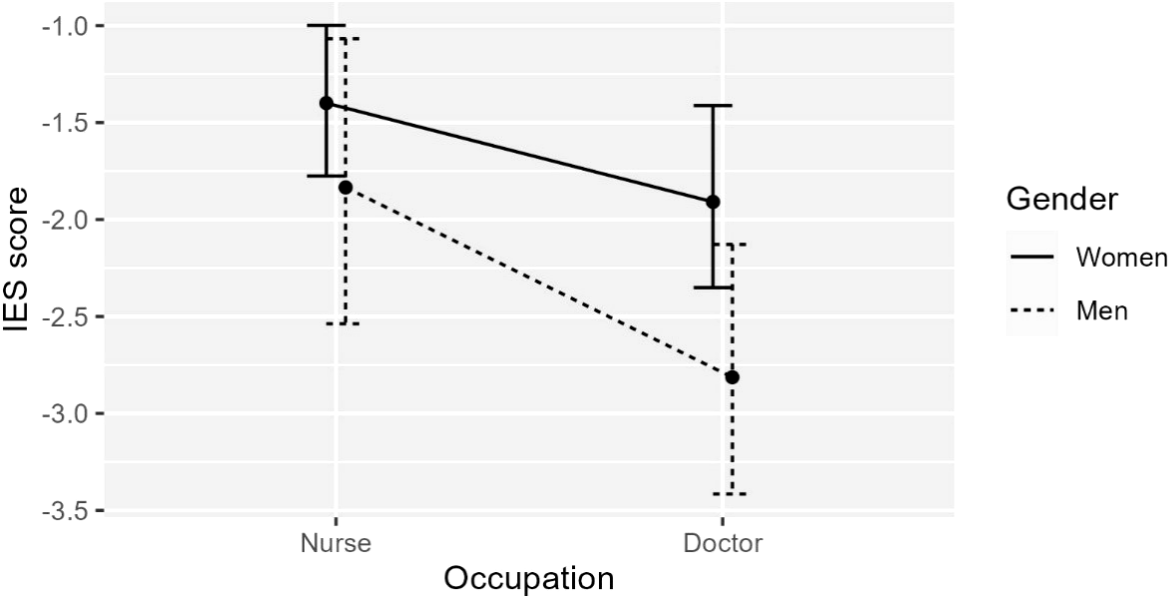
The effect of Gender (women/men) and Occupation (doctor/nurse) on IES-R score. Both predictors stat. sig., but no statistically significant interaction.

*IES-R*. Both gender and occupation were statistically significant predictors of IES-R score (**Figure 2**, see **Table 7**, Panel 2, Model 1), *F*(2,142) = 6.986, *p* <.001, adj. *R* ^2^ = .077: men had statistically significant lower IES-R scores than women, *b* = -0.647, *t*(142) = -2.301, *p* <.05; doctors had stat. sig lower IES-R scores than nurses, *b* = -0.689 *t*(142) = -2.509, *p* <.05 (note that higher IES-R scores represent higher risk of PTSD).

**Figure 3:**
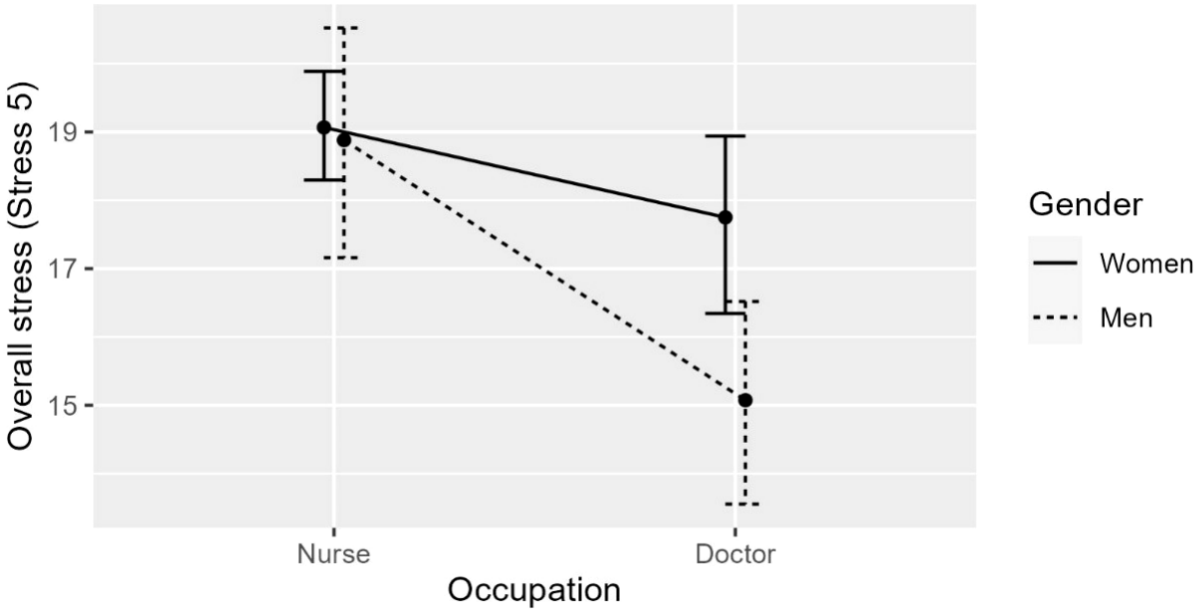
The effect of Gender (women/men) and Occupation (doctor/nurse) on overall stress. Both predictors stat. sig., but no statistically significant interaction.

*Overall stress (Stress 5)*. both gender and occupation type were statistically significant predictors of overall stress (**Figure 3**), *F*(2,142) = 9.543, *p* <.001, adj. *R* ^2^ = .106: men reported statistically significantly lower levels of overall stress than women, *b* = -1.312, *t*(142) = -1.977, *p* <.05; doctors reported statistically significant lower levels of overall stress as nurses, *b* = -2.267, *t*(142) = -3.500, *p* <.001.

### 3.5 Hypothesis 4: the relationship of anxiety and post-traumatic stress with COVID-specific stress

**Figure 4:**
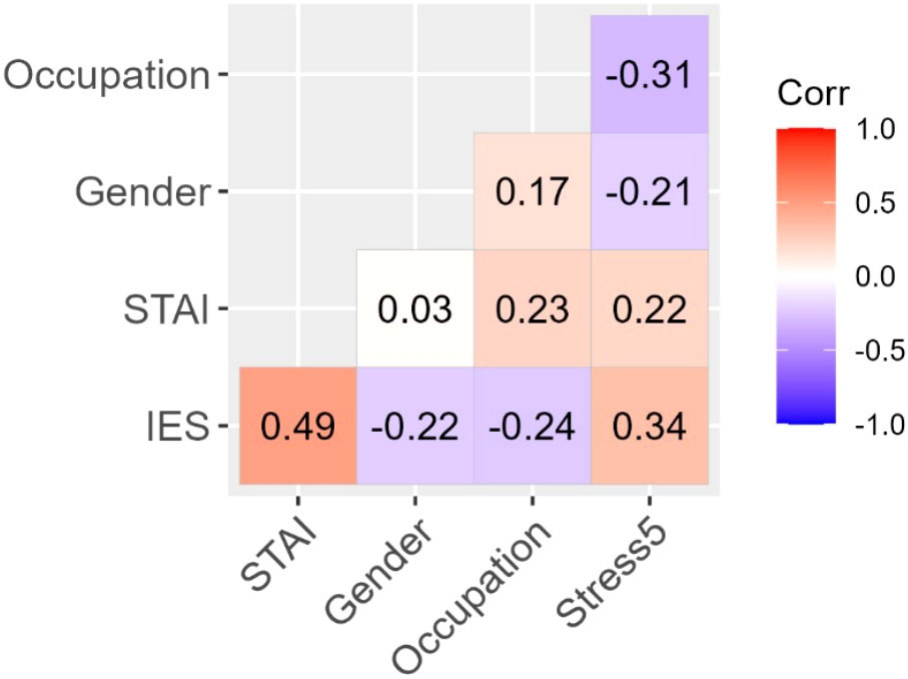
Correlations of gender and occupation categories with anxiety, stress, and PTS. Each *r* is statistically significant at *p* <.05 except for Gender/STAI-S. Fields between binary variables (Gender and Occupation) and interval-level variables are interpreted as point-biserial correlations (negative values indicate lower values for women and nurses, respectively). The Gender/Occupation field has no substantive meaning.

To test the effect of COVID-specific stress (measured by Stress 5) on anxiety and post-traumatic stress (PTS), we conducted two hierarchical regression analyses with STAI-S and IES-R as outcome variables, respectively. To control for effects of gender and occupation, the first level of hierarchy in both cases included gender (coding: women = 0, men = 1) and occupation (coding: nurse = 0, doctor = 1), then we added the effect of Stress 5 at the second level of hierarchy. The correlation coefficients between the variables are presented in **Figure 4**. The results of the regression analyses are summarized in **Table 7**.

In both cases, the model containing Stress 5 was a statistically significant better fit than the baseline model including gender and occupation as predictors. In the STAI model, occupation had a relatively stronger effect than Stress 5. However, in the IES-R model, including Stress 5 rendered the effects of gender and occupation statistically non-significant. The findings highlight the importance of COVID-specific stress factors on anxiety and PTS while controlling for the effects of differences due to gender (men/women) and occupation (nurse/doctor).

### 3.6 Hypothesis 5: differences between nurses and doctors in coping strategies

**Figure 5:**
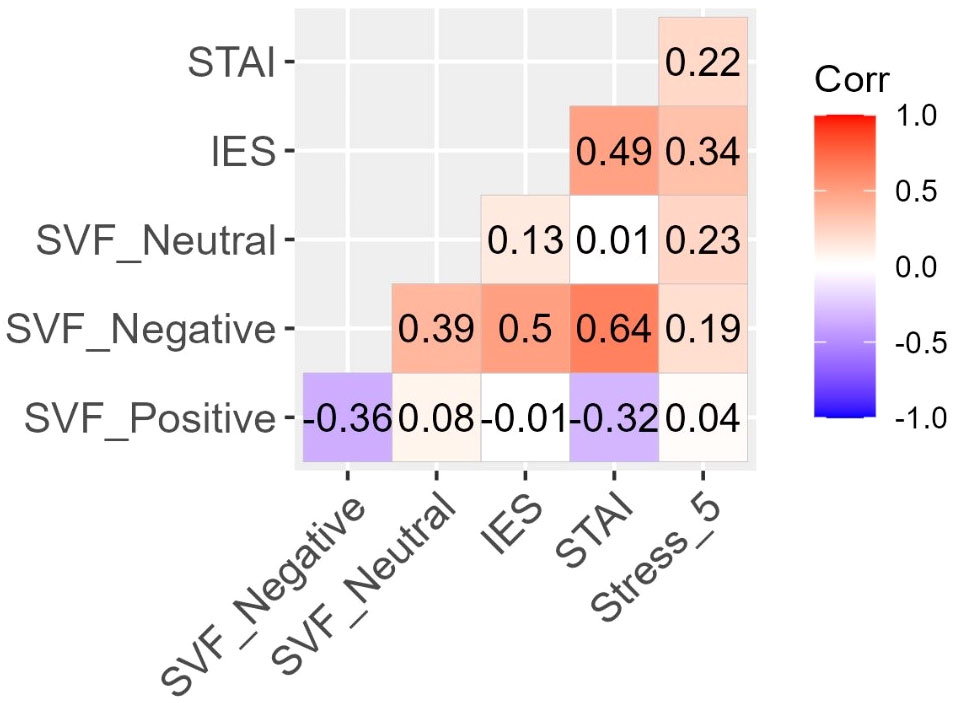
Correlations of psychological coping strategies (positive, negative, and neutral) with measures of mental duress (anxiety, stress, and PTS). Positive strategies were only statistically significant related to STAI-S (*r* = -.32, *p* <.01). Negative strategies were statistically significant positively related to STAI-S (*r* = .64, *p* <.01), IES-R (*r* = .50, *p* <.01) and Stress 5 (*r* = .19, *p* <.05). Neutral strategies were only statistically significant related to Stress 5 (*r* = .23, *p* <.01).

The descriptive statistics of psychological coping strategies across occupation and gender are presented in **Table 8**. The correlations between positive, negative, and neutral psychological coping strategies with outcome measures of mental duress (STAI-S, IES-R, and Stress 5) are presented in **Figure 5**.

**Table 8:** Descriptive statistics of SVF sub-scales across occupation (nurse/doctor) and gender (women/men).

Occupation	Gender	*n*	Positive Mean	Positive *SD*	Negative Mean	Negative *SD*	Neutral Mean	Neutral *SD*
Nurse	Women	61	2.29	0.49	1.52	0.74	2.27	0.57
	Men	25	2.13	0.35	1.30	0.73	1.90	0.54
Doctor	Women	32	2.03	0.50	1.91	0.81	2.22	0.45
	Men	27	2.08	0.40	1.43	0.90	1.92	0.61

Positive coping strategies included the following seven SVF dimensions: minimization/downplaying, denial of guilt, substitute gratification, situation control, reaction control, positive self-instructions, and distraction. Positive coping strategies were statistically significant negatively related to STAI-S with medium effect size (*r* = -.32, *p* <.01, medium effect size); however, they were not related to IES-R and Stress 5.

Negative coping strategies included the following four SVF dimensions: escape, rumination, resignation, and self-blame. Negative strategies were associated with increased level of stress (STAI-S, *r* = .64, *p* <.01, large), PTS (IES-R, *r* = .50, *p* <.01, large), and COVID-specific stress (Stress 5, *r* = .19, *p* <.05, small).

Neutral coping strategies included two SVF dimensions: need for social support and avoidance. Neutral strategies were only statistically significant correlated with COVID-specific stress (Stress 5, *r* = .23, *p* <.01, small).

We tested the mean difference in each coping strategy across gender (women/men) and occupation (nurse/doctor). Both predictors were coded as binary antecedents with nurse and women as base categories (nurse = 0, doctor = 1; women = 0, men = 1), and the interaction of the antecedents were included in each model. This approach is equivalent to a 2x2 factorial ANOVA, where the reported regression coefficients are interpreted as mean differences between the groups attributable to the grouping variable (positive values indicate higher value for men and doctors) while controlling for the other variable and the interaction effect. Only statistically significant effects are reported.

For positive coping strategies (*F*(3,141) = 2.713, *p* <.05, adj. *R* ^2^ = .034), only the effect of occupation was stat. significant, *b* = -1.529, *t*(141) = -2.553, *p* <.05; on average, nurses used more positive coping strategies than doctors with a small effect size (*r* = .210). For negative coping strategies (*F*(3,141) = 3.307, *p* <.05, adj. *R* ^2^ = .046), only the effect of occupation was stat. significant, *b* = 2.318, *t*(142) = 2.250, *p* <.05; on average, doctors used more negative coping strategies than doctors with a small effect size (*r* = .186). For neutral coping strategies (*F*(3,141) = 4.483, *p* <.01, adj. *R* ^2^ = .068), only the effect of Gender was stat. significant, *b* = -2.251, *t*(142) = -2.885, *p* <.01; on average, women used more neutral coping strategies than men with a small effect size (*r* = . 236).

### 3.7 The role of protective factors in coping with anxiety and post-traumatic stress

Overall, nurses and doctors tended not to differ in the extent to which they agreed with the five protective factors. The lowest level of agreement was for employer support (nurses: *M* = 2.24, *SD* = 1.13, doctors: *M* = 2.73, *SD* = 1.19) and work-life balance (nurses: *M* = 2.47, *SD* = 1.24, doctors: *M* = 2.70, *SD* = 1.21). Moderate to high agreement was found for support from close environment (nurses: *M* = 4.25, *SD* = .93, doctors: *M* = 4.37, *SD* = .96), liking one’s job (nurses: *M* = 3.66, *SD* = 1.30, doctors: *M* = 4.05, *SD* = 1.08), and thinking of doing a good job (nurses: *M* = 4.56, *SD* = .71, doctors: *M* = 4.03, *SD* = .92).

To explore the role of protective factors (see **Table 1**) in coping with anxiety and PTS, we calculated correlation coefficients between each protective factor and STAI-S and IES-R, respectively, separately for nurses and doctors, and tested the difference in correlations between the occupation categories (**Table 9**).

**Table 9:** Correlations between protective factors, and anxiety (STAI-S) and PTS (IES-R) for nurses and doctors.

	Criterion: STAI-S				Criterion: IES-R			
Protective item	Nurse	Doctor	*z* diff.	*p* diff.	Nurse	Doctor	*z* diff.	*p* diff.
Protect 1 (support by employer)	-.28**	-.13	-0.91	.18	-.24*	-.44**	1.32	.19
Protect 2 (support by people)	-.10	-.34**	1.47	.07	.06	-.35**	2.46	.014
Protect 3 (likes job)	-.21	-.58**	2.60	.005**	-.26*	-.44**	1.19	.23
Protect 4 (does good job)	-.13	-.55**	2.82	.002**	-.07	-.35**	1.71	.09
Protect 5 (work/life balance)	-.44**	-.46**	0.15	.44	-.40**	-.53**	0.96	.34

Parametric statistical inference for differences is based on *z*-transformed *r* values [see (42)]. Bonferroni-corrected alpha threshold for a family of five tests: *p* = .01. See **Table 1** for item wordings.

For coping with anxiety, liking one’s job (Protect 3) and feeling like doing a good job (Protect 4) were statistically significant factors for doctors, but not for nurses (both correlations were statistically significant different between occupation groups). Conversely, support by the employer (Protect 1) was a statistically significant protective factor for nurses, but not for doctors. Work-life balance (Protect 5) was important for both doctors and nurses.

For PTS, support by people (Protect 2) was a protective factor for doctors, but not for nurses (the difference between correlations approached stat. significance); the same can be observed for feeling like doing a good job (Protect 4). Just as for anxiety, work-life balance (Protect 5) was negatively related to PTS in each occupation group.

## 4 Discussion

Numerous studies have underscored the profound adverse impact of the COVID-19 pandemic on the mental health of HCW (1–3,11,43). To gain a comprehensive understanding of the evolving state of HCW’s mental health in subsequent waves of the pandemic, we conducted analyses to ascertain whether variables such as anxiety, post-traumatic stress, COVID-specific stress, and PTSD were influenced by factors such as ward affiliation, occupation, and gender. Furthermore, our investigation delved into the roles played by protective factors and coping strategies in shaping these outcomes.

Hypothesis 1, suggesting that the mental health of HCW varies depending on ward affiliation, could not be substantiated. No significant differences were observed between wards in terms of anxiety, post-traumatic stress, and COVID-specific stress. Additionally, ward affiliation was not associated with the proportion of individuals exhibiting PTSD symptoms. However, earlier research on the mental health of HCW during the initial phases of the pandemic yielded contrasting findings. Specifically, a significant correlation was established between ward affiliation, used as an operationalization of the severity of cases, and mental health outcomes. This was observed not only for the COVID-19 pandemic (3, 12) but also for prior pandemics (10). Given that concerns about infecting oneself (1, 3) and transmitting the virus to close family members (3, 8) were highlighted as potent stressors, it’s noteworthy that by the third year of the pandemic, the perceived risk of infection had diminished due to several contributing factors. These include the availability of vaccinations, personal experience with a prior COVID-19 infection, and the implementation of new protocols within healthcare facilities. Kałucka et al. (47) demonstrated that the primary motivations behind HCW choosing to be vaccinated were centered on safeguarding their own health and the well-being of their families. Furthermore, insights from a two-wave study conducted among the general population in Germany (April 2021 and August/September 2021) supported the notion that fear reduction associated with vaccination was more pronounced compared to fear reduction without vaccination (48). Additionally, the process of habituation, coupled with a growing familiarity with the progression of COVID-19 infections, could potentially contribute to the mitigation of stress levels associated with these factors. Notably, the German two-wave study (48) highlighted a decrease in fear related to COVID-19 even in the absence of vaccination, further emphasizing the potential role of habituation in alleviating mental stress. Given that further analyses highlight the enduring significant impact of COVID-specific stressors, such as the fear of infection and the potential to transmit the virus to others, on the mental health of HCW, other or more complex explanations seem appropriate. First, we assume that HCW with pre-existing conditions or with vulnerable relatives exist randomly in all ward affiliations. Furthermore, research showed that in the general population HCW were stigmatized and avoided for fear of infection, in earlier pandemics as well as during the COVID-19 pandemic (49). To be stigmatized leads to stress (50) and partly, negative attitudes towards oneself are internalized (51). So, the feeling to be infectious may remain strong even after being vaccinated or having gone through an infection. Additionally, it is noteworthy that while vaccination provides robust protection against severe infection, its effectiveness against milder or asymptomatic cases is comparatively limited (52). Equally relevant, the other dimensions of COVID-specific stress apply universally to HCW across all wards. For instance, the fear of the pandemic enduring over an extended period was substantiated at that time, given the prominence of the delta variant and the emerging omicron variant. Notably, there was a lack of signs indicating a decline in the pandemic’s impact despite an increasing percentage of vaccinations (53, 54). Likewise, concerns about nosocomial infections (55, 56), the postponement of essential surgeries (57), and resource shortages (36) have repercussions for all HCW across different wards, intensifying concerns about patients. Moreover, the enduring high workload throughout the prolonged pandemic has also taken a toll on HCW and their mental health (53).

In relation to the disparities observed among different occupational groups (Hypothesis 2), our findings revealed that doctors, at that point of time, exhibited higher levels of anxiety in comparison to nurses. Conversely, nurses reported greater instances of post-traumatic stress and COVID-specific stress compared to doctors. Notably, the odds of developing post-traumatic stress disorder (PTSD) were 3.28 times higher for nurses than for doctors. These outcomes align with the majority of prior research conducted during the early stages of the pandemic, which consistently indicated elevated mental stress levels among nurses (3, 11–14). Specifically, this effect was most pronounced in the context of post-traumatic stress scores (10, 20). But there are hints that this is also valid for anxiety (1). Conversely, our findings diverge from the outcomes reported by Pei et al. (6), who discovered no discernible differences in terms of occupational groups regarding mental stress during the third year of the pandemic. Notably, their study encompassed HCW from square cabin hospitals in China, distinct entities designated for the exclusive treatment of a notably elevated volume of COVID-19 cases. This unique context likely presents distinct challenges and an augmented mental burden compared to conventional healthcare facilities. It is plausible that the conditions in these isolated structures contribute to a universally elevated level of mental stress across all groups of HCW (6). However, our Hypothesis 2 is to be accepted only in parts since doctors here suffered from a higher state anxiety than nurses. To understand this result better, analyses with a split doctor category are to be considered. Altogether, these results identify the subcategory medical specialist as most burdened group of HCW in that later stage of the pandemic, since they show high values in all three mental stresses, COVID-specific, anxious, and post-traumatic stress. In comparison, nurses suffer from the highest levels of COVID-specific and post-traumatic stress, but not anxiety, and residents suffer from high anxiety and COVID-specific stress, but not post-traumatic stress. Only senior doctors show in all three stress measures relatively low levels. Seeing this, we conclude that not only proximity to patients, but also job status linked with high responsibility is crucial for mental stress of HCW at this time point of the pandemic. Medical specialists are uniquely positioned as they operate in close proximity to patients while also shouldering a significant burden of responsibility. Consequently, gauging the mental burden of HCW necessitates a more intricate framework than solely focusing on occupational status, ward, or gender. To unveil the underlying reasons for the observed disparities in anxiety, especially concerning medical specialists, qualitative methodologies such as interviews could offer valuable insights for future research.

Regarding the difference between gender (Hypothesis 3), we found no association between gender and the proportion of people with PTSD. Since occupation and gender could be confounded due to a high proportion of women amongst nurses (1), multiple regression analyses were calculated to evaluate possible interactions between gender and occupation. Occupation was predictor for all three, anxiety, post-traumatic stress, and COVID-specific stress. Whereas gender was only predictor for post-traumatic stress and COVID-specific stress. Further, no interaction effects could be discovered. The results regarding post-traumatic and COVID-related stress go in line with prior research on early stages and corroborate that women suffer more from this type of mental stress (1, 10, 19). Kramer et al. (1), concluded this also for anxiety, whereas in the here presented study, doctors and especially medical specialists were the most affected group (see above). Since explanations for the here found occupation differences are thought to lie in COVID-specific stress factors, we analyzed not only the question, which factors are influencing mental stresses during the later stage of the pandemic, but we also asked which influence do have COVID-specific stress variables in comparison to gender and occupation on anxiety and post-traumatic stress (Hypothesis 4). Therefore, hierarchical multiple regressions were conducted with two models. One model checked the influence of gender and occupation and the other model included as predictor also COVID-specific stress. For both, anxiety and post-traumatic stress, the model including COVID-specific stress fitted better. For anxiety, occupation and COVID-specific stress were predictors, whereas for post-traumatic stress only COVID-specific stress remained predictor in the better model. These findings highlight the importance of COVID-specific stress as factor for anxiety and post-traumatic stress while controlling for the effects of differences due to gender and occupation. The data confirm our Hypothesis 4 and show that in the later stage of the pandemic, main COVID-specific predictors for mental stress remain the same. This is since COVID-specific stress here is defined by an aggregation of best COVID-related predictors reported in the literature on the early stages (worry about own health (1, 3): fear of infecting others (3, 8): more concerned about patients (35, 36): overwhelming current workload (1): fear of continuation of pandemic (anticipated load expectation)). The reasons for ongoing COVID-specific stress were discussed above for Hypothesis 1. Occupation as strong predictor of anxiety results from a high level of anxiety in medical specialists and resident doctors (see above, Hypothesis 2).

Since coping strategies (23) and protective factors (22, 24) have shown the potential to influence mental health of HCW in earlier stages of the pandemic, this influence was investigated also here.

In accordance with Labrague’s (23) findings, HCW in this study employed more positive than negative coping strategies. Therefore, even during the later stages of the pandemic, HCW had continued access to psychological protective mechanisms akin to those evident in earlier phases (23). Notably, our findings also revealed that the utilization of negative coping strategies yielded the most pronounced adverse impact on measures of mental distress. In this context, the empirical data effectively affirm Hypothesis 5. Although doctors tended to use more negative coping strategies on average than nurses, the size of this effect was small. There were no gender differences in the use of negative strategies. The use of positive coping strategies was related to lower anxiety with medium effect size. Nurses tended to use more positive strategies than doctors, but the difference was small; again, there was no difference between men and women. Regarding positive coping strategies, Hypothesis 5 was confirmed only in parts since it is valid only for anxiety. Neutral coping strategies were associated with slightly larger levels of COVID-specific stress; on average, women used slightly more neutral strategies than men, with no difference between nurses and doctors. So, also in later stages of the pandemic coping strategies are important in predicting mental duress, particularly, negative strategies increased all three measures of post-traumatic stress, anxiety, and COVID-specific stress. This finding is in line with theoretical and empirical background of the measurement where higher scores in negative coping strategies go together with worsening stress levels (32) and with literature (23). Whereas Erdmann and Janke (32) and Labrague et al. (23) report that also positive coping strategies reduce various mental stresses, this is valid here only for anxiety. Further, since nurses used slightly more positive strategies and doctors had higher levels of anxiety, prevention measures and coping training could be inferred from this finding, specifically more tailored to groups of concern. For instance, train doctors in employing more positive coping strategies. In terms of gender, prior research showed also that women used more the neutral strategies need for social support (58, 59) and avoidance (59). The reported (58, 59) gender differences for men using more positive, and women using more negative strategies we did not find. Overall, the variations attributed to gender or occupation were minimal. The findings from hierarchical multiple regressions conducted for Hypothesis 4 underscored that COVID-specific stress was the main predictor of mental stress of HCW in the later stage of the pandemic, so we suppose that other factors are not that strong in the light of the burdensome influence of COVID-specific stress. It should be noted that HCW with the highest propensity of employing avoidance strategies could be lost for this research due to their likelihood of having left their positions. Kramer et al. (1) reported heightened job-quitting of HCW during the pandemic.

The results of the exploratory investigation into the role of protective factors can be summarized as follows: overall, support from employers and achieving a work-life balance received the lowest agreement scores among the categories, while the remaining three categories garnered medium to high scores, particularly liking one’s job and thinking doing a good job. Notably, doctors appear to benefit from a multitude of protective factors (9 out of 10), with only support from employers not being a protective factor for anxiety. In contrast, nurses exhibit fewer protective factors associated with anxiety or post-traumatic stress (5 out of 10). Furthermore, correlations between protective factors for nurses were generally small, in contrast to doctors, except for the domain of work-life balance. Prior research at the begin of the pandemic showed that nurses had lower levels of protective factors, e.g., Lin et al. (24),. Altogether, results show that also for the later stages of the COVID-19 pandemic, similar protective factors (professional identity, collegial support, supportive leaders, self-care) as in prior pandemics and earlier in the COVID-19 pandemic are effective (22), wherein important occupational differences are to be considered. However, it is crucial to account for significant occupational differences. The high-anxiety doctor groups identified in this study (specialists and residents) could benefit from accessing the established protective factors that have demonstrated effectiveness. Similarly, nurses, experiencing higher anxiety and post-traumatic stress, have limited access to protective factors. Interestingly, they do experience benefits from protective factors that they themselves rate lower (support from employers and work-life balance). Consequently, it becomes imperative to enhance these protective factors during the post-pandemic phase and future pandemics alike. Qualitative research should be considered to delve into the reasons behind the relatively fewer protective factors among nurses and whether other effective protective factors exist. Employers of HCW should prioritize work-life balance, especially since it emerges as the most potent protective factor for both nurses and doctors during this stage of the pandemic.

### 4.1 Limitations

In general, it should be noted that the data presented in this study rely on self-reported responses obtained through an online survey. Consequently, potential self-selection processes might hold significance, as online surveys inherently carry a risk of selection bias. HCW may opt to participate in an attempt to either downplay or emphasize the current situation. Notably, the relatively low prevalence of mental health issues reported by senior doctors warrants attention. Given the small number of high-ranking doctors and the potential exposure of personal identifiers in the dataset due to other socio-demographic variables, these individuals may have responded by denying mental health challenges. Additionally, the study design is cross-sectional, which limits the capacity to track changes within the same HCW across different time periods. Instead, comparisons are reliant on existing literature findings. Furthermore, the sample utilized in this study is not fully representative, as it exclusively encompasses German-speaking HCW from a single hospital. The advantage of monocentric studies lies in the creation of comparable working conditions among participants. Furthermore, it’s worth mentioning that this study constitutes a complete survey, which significantly expands its reach and coverage. In general, response rates about 20 percent are normal (60). Moreover, such studies serve as informative sources for devising protective strategies for HCW in future pandemics. As previously mentioned, HCW who have exited their jobs are not accounted for in this survey; however, they could contribute crucial insights into the mental health landscape of HCW. This group should be the subject of meticulous investigation in future research. Finally, the relatively modest participant count may limit the depth of further analyses. This limitation should be addressed in future quantitative research designs.

## 5 Conclusions

In summary, our findings show that HCW are still suffering from mental stress in the third year of the pandemic. The suffering is influenced by similar factors as it is described in literature for earlier phases. Differences to earlier phases we found in ward affiliation which is no longer a variable of concern for explaining differences in mental health of HCW. Consequently, all HCW within a hospital warrant equal attention and safeguarding against mental stress. Further, even if nurses are the occupational group with the highest mental stress as in prior research, detailed analyses show that medical specialists with closeness to the patients and high level of responsibility are the most burdened group in total, since they suffer in addition to high post-traumatic and COVID-specific stress from high state anxiety contrary to nurses. So, for most effective countermeasures, other detailed analyses of groups of concern are appropriate than occupation or ward affiliation. Analyses showed further that COVID-specific stress proved as the strongest predictor of mental stress in HCW in this point of time, wherein COVID-specific stress factors remain the same as reported in literature on the early phases of the pandemic. HCW proved here to have still access on coping strategies using more positive than negative strategies like in prior phases. Negative strategies increased mental stress, whereas positive strategies alleviated only anxiety. Regarding protective factors we found that doctors benefited from many protective factors while nurses had only access to fewer protective factors associated with anxiety or post-traumatic stress which is similarly reported for earlier waves. These findings may have implications for developing protection strategies for the post-pandemic period as well as for potential future pandemics. To mitigate the prolonged mental health impact on healthcare workers (HCW) due to the COVID-19 pandemic, strategies should include providing immediate access to mental health services, fostering resilient coping mechanisms, creating supportive work environments, advocating for mental health-friendly policies, tailoring interventions to meet the diverse needs of HCW, and continuously monitoring and adapting mental health support programs. These efforts must be collaborative, involving healthcare institutions, policymakers, and the community. Finally, future research should explore the long-term effects of the pandemic on HCW’s mental health and the effectiveness of various support and intervention strategies.

## Data availability statement

The raw data supporting the conclusions of this article will be made available by the authors, without undue reservation.

## Ethics statement

The studies involving humans were approved by Ethics Commission of the Faculty of Psychotherapy Science and the Faculty of Psychology of the Sigmund Freud University Vienna (Reference: UBRPHK9TAQOGJ888066). The studies were conducted in accordance with the local legislation and institutional requirements. The participants provided their written informed consent to participate in this study.

## Author contributions

CE: Conceptualization, Writing – original draft, Writing –review & editing, Methodology, Project administration, Supervision.RS: Conceptualization, Writing – original draft,Writing – review & editing, Methodology.PA: Data curation, Investigation.GA: Data curation, Formal Analysis, Methodology, Writing – original draft, Writing – review & editing.KH: Conceptualization, Resources, Supervision, Validation, Writing – review & editing.

## Footnotes

1 Items corresponding to sub scales: Intrusion: 1, 3, 6, 9, 14, 16, 20; Avoidance: 5, 7, 8, 11, 12, 13, 17, 22; Hyperarousal: 2, 4, 10 ,15, 18, 19, 21. Formula to aggregate total IES score: -0.02 x Intrusion + 0.07 x Avoidance + 0.15 x Hyperarousal - 4.36.

2 Unstandardized regression coefficients indicate the unit of change in the outcome associated with each predictor while holding the other predictor(s) constant.
